# Spatiotemporal Characteristics of Ecological Conditions and Its Response to Natural Conditions and Human Activities during 1990–2010 in the Yangtze River Delta, China

**DOI:** 10.3390/ijerph15122910

**Published:** 2018-12-19

**Authors:** Ziqi Meng, Min Liu, Qiannan She, Fang Yang, Lingbo Long, Xia Peng, Ji Han, Weining Xiang

**Affiliations:** 1 Shanghai Key Lab for Urban Ecological Processes and Eco-Restoration, School of Ecological and Environmental Sciences, East China Normal University, Shanghai 200241, China; zqmeng_mzq@163.com (Z.M.); sheqiannan.Lindas@163.com (Q.S.); fangyang.franny@gmail.com (F.Y.); lird_long@126.com (L.L.); claire_px@126.com (X.P.); jhan@re.ecnu.edu.cn (J.H.); 2 Institute of Eco-Chongming (IEC), Shanghai 200062, China; 3 Library of East China Normal University, Shanghai 200241, China; 4 Center for Ecological Wisdom and Practice Research, College of Architecture and Urban Planning, Tongji University, Shanghai 200092, China; wxiang@uncc.edu

**Keywords:** ecological conditions, natural conditions, human activities, Lindeman-Merenda-Gold, Yangtze River Delta

## Abstract

The Yangtze River Delta (YRD) region, including Shanghai City and the Jiangsu and Zhejiang Provinces, is the largest metropolitan region in China. In the past three decades, the region has experienced an unprecedented process of rapid and massive urbanization, which has dramatically altered the landscape and detrimentally affected the ecological environments in the region. In this paper, we analyzed the spatiotemporal variations of ecological conditions (Eco_C) via a synthetic index with analytic hierarchy processes in the YRD during 1990–2010. The relative contributions of influencing factors, including two natural conditions (i.e., elevation (Elev) and land-sea gradient (Dis_coa)), three indicators of human activities (i.e., urbanization rate (Urb_rate), per capita GDP (Per_gdp), the percentage of secondary and tertiary industry employment (Per_ind)), to the total variance of regional Eco_C were also investigated. The results showed that: (1) The Eco_C over YRD region was “Moderately High”, which was better than the national average and demonstrated obvious spatial variations between south and north. There existed fluctuations and an overall increasing trend for Eco_C during the study period, with 20% of the area being deteriorated and 40% being improved. (2) The areas with elevation below 10 m was relatively poor in Eco_C, while the regions above 1000 m showed the best Eco_C and had the most obvious changes (9.33%) during the study period. (3) The selected five influencing factors could explain 91.0–94.4% of the Eco_C spatial variability. Elevation was the dominant factor for about 42.4–52.9%, while urbanization rate and per capita GDP were about 32.5% and 9.3%.

## 1. Introduction

According to the World Urbanization Prospects, 55% of the world’s people live in urban areas in today’s world [1]. Urban growth induces the modification of natural land cover and the biophysical environment [2,3], which significantly influences the local and global ecosystems and the services they provide to human and other life on earth [4,5]. China, as a developing country and a major emerging economy in the world, had been urbanizing at a record speed with rapid economic development during the past three decades, which had also led to a range of environmental issues [6]. As one of the largest metropolitan regions in China, the Yangtze River Delta (YRD) has experienced an unprecedented process of rapid and massive urbanization, which has dramatically altered the landscape with high population density [7]. Analyzing the ecological conditions (Eco_C) and its response to natural conditions and human activities are of extreme importance to the YRD with respect to the conservation of natural resources and sustainable development [8].

Ecological conditions refer to the state of ecological systems, which includes their physical, chemical, and biological characteristics and the processes and interactions that connect them. It can provide the essential conditions for human survival and socioeconomic development within a certain time and space [9]. With the massive urbanization and intensive industrialization, there appeared myriad environmental problems which are encountered in many developing countries today and in many developed countries several decades ago. The growth of cities and towns together with the associated increase in their ecological footprint are the most serious ecological problems facing the world [10]. Improving human well-being and preserving the ecological conditions within and beyond urban areas is a great challenge [4]. Thus, understanding the variation patterns of ecological conditions during the past and present at both local and global scales is necessary to address sustainable development and to avoid negative consequences [11]. However, many previous studies on regional ecological conditions were usually based on a single indicator related to the natural environment, such as the land surface temperature (LST) [12,13], land use and land cover change (LUCC) [14] and the Normalized Difference Vegetation Index (NDVI) [15]. Due to the complexity of ecological conditions with various spatio-temporal scales and increasing in human disturbances, a comprehensive index combined with indicators for different aspects of ecological conditions could help us reach an objective understanding of regional ecological conditions [16,17,18].

According to previous studies, accompanying the rapid development of an economy and urbanization, tremendous changes have taken place in land use. As a result of the expansion of built-up areas, the demand for housing, commercial, and industrial infrastructure is often satisfied through land use changes to exploit natural lands such as arable land, forests, water bodies, and open spaces [19]. In addition to these effects, changes in land use, especially the increase of urban construction, can damage the ecological conditions including the climate, soil, and biological components. Due to excessive exploitation and utilization of land resources, the ecological conditions in many places have been destroyed, which seriously threatens ecological security [20]. Meanwhile, as people pay more and more attention to eco-environmental problems, governments have taken measures to protect ecological conditions and reduce pollution, such as policies of comprehensive control over construction land and the ecological civilization campaign in China [21]. Due to these analyses, previous studies had mostly concentrated on the relationship between urbanization and Eco_C at the local [2], regional [3] and global scales [22]. For example, Huang and Fang (2003) had argued that an interactive mandatory relationship existed between Eco_C and urbanization [23]. Yi et al. (2018) established a comparative evaluation method of positive and negative ecological elements to understand the impact of human activities on coastal ecosystems [24]. In addition, the influence of urbanization on global environmental changes, whether LUCC [25], water resources [26], emission of pollutions [27], energy use [28] and ecosystem-service values [29], have been analyzed qualitatively and quantitatively. Whereas few studies have reported the influence of natural conditions on Eco_C. In fact, the ecological conditions at a regional scale were influenced by many natural factors such as climate and environmental change [30,31], elevation differentiation [32] and land-sea gradients [33]. However, natural conditions and human activities have rarely been considered simultaneously when investigating the influencing factors for spatiotemporal variations of ecological conditions. 

Building on the above analysis, in this paper, we aimed to investigate the spatiotemporal variation characteristics of ecological conditions in the YRD and reveal their responses to natural conditions and human activities. Through the Technical Criterion for Ecosystem Status Evaluation (HJ192-2015) (CRITERION) [34], published by the Ministry of Environmental Protection of the People’s Republic of China, we analyzed the spatiotemporal variations of ecological conditions (Eco_C) via a synthetic index with analytic hierarchy processes in YRD during 1990–2010 at the pixel scale, based on regional land-use data, remote sensing data and related pollution information. Besides this, we evaluated the potential role of natural conditions (i.e., elevation (Elev) and land-sea gradient (Dis_coa)) and local anthropogenic activities (i.e., urbanization rate (Urb_rate), per capita GDP (Per_gdp) and the percentage of secondary and tertiary industry employment (Per_ind)) on the spatiotemporal patterns of Eco_C in the YRD. Moreover, the relative contributions of influencing factors, including two natural conditions and three indicators of human activities, to the total variance of regional Eco_C were also investigated.

## 2. Materials and Methods

### 2.1. Study Area

**Figure 1:**
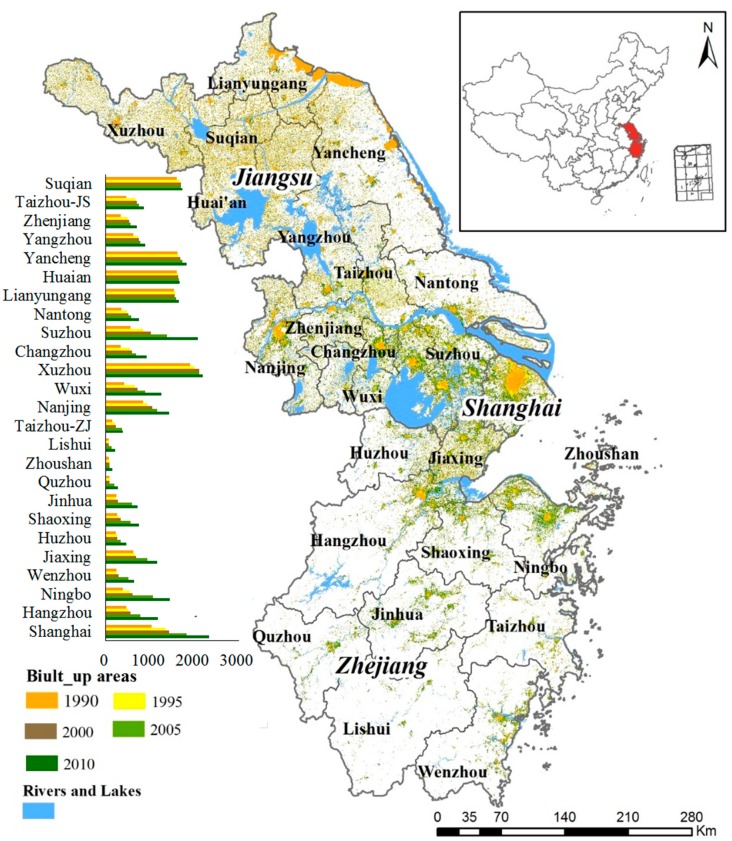
The geographical location of the Yangtze River Delta (YRD) in China. The expansion of built-up areas during 1990–2010 was also illustrated.

The Yangtze River Delta (116°29′–123°25′ E, 27°14′–35°33′ N), covering a total area of approximately 210,700 km^2^, is located at the junction of the Yangtze River and the East China Sea (Figure 1). It encompasses Jiangsu province, Zhejiang province, and the Shanghai municipality, with 25 cities in total. Under the influence of the East Asian monsoon, the YRD region has an annual mean temperature of 17.5 °C and receives 1671 mm of annual precipitation [35,36]. The northern areas of the YRD are plains, while the rest of the territory contains a complex topography comprised of hills, and low and medium-high mountains. As the engine of China’s economic development, the region has experienced an unprecedented rate of rapid and massive urbanization, which dramatically altered the landscape and detrimentally affected the ecological conditions in the region [37]. The region’s population has increased from 123.4 million in 1990 to 160.1 million in 2016, with energy consumption increasing from 114.3 million tons of coal equivalent (Mtce) to 630.4 Mtce during the same period. In 2016, its gross domestic product (GDP) reached CNY 15.3 trillion (USD 2290.0 billion), contributing 20.5% of the country’s GDP [35,36].

### 2.2. Evaluation of Ecological Conditions

In this study, the ecological conditions in the YRD region were evaluated with an ecological index (EI), a synthetic index provided by the “Technical Criterion for Ecosystem Status Evaluation (Trial) (HJ192-2015)” (CRITERION) announced by the Ministry of Environmental Protection of the People’s Republic of China (MEP) [34]. According to actual conditions in the YRD, we made some revisions when we applied the HJ192-2015. In the criterion HJ192-2015, EI is composed of the Biological Abundance Index (BAI), Vegetation Cover Index (VCI), Water-net Density Index (WDI), Land Deterioration Index (LDI), Pollution Load Index (PLI) and the Environmental Restriction Index (ERI). The CRITERION considered that regions with high vegetation cover, abundant biodiversity as well as stable ecosystems could have high quality. The higher the EI value, the better the ecological conditions. Previous studies [38] have indicated that there existed a negative correlation between land degradation and NDVI, which implied that the higher the NDVI, the lower the degree of land degradation. Therefore, LDI was characterized by the annual maximum value of NDVI (NDVI_max_) in this paper. Since NDVI_max_ has the function of characterizing the degree of regional land degradation and vegetation coverage, we used NDVI_max_ as the description of VCI and LDI. Besides this, in the criterion HJ192-2015, the Pollution Load Index (PLI) was determined by many pollutants such as chemical oxygen demand (COD), ammonia nitrogen, sulfur dioxide (SO_2_), soot, nitrogen oxides, solid waste and total nitrogen, while the Environmental Restriction Index (ERI) had not been quantitatively evaluated. Therefore, considering the availability of long-term data, SO_2_, COD and solid waste were used to comprehensively characterize the PLI and ERI in this paper, namely the Pollution Load Index (PLI). Ultimately, in this paper, we selected four sub-indexes, including BAI, VCI, WDI and PLI to reflect the ecological conditions, and the weight of each factor was adjusted according to the actual conditions, as shown in Table 1. There were five levels to evaluate Eco_C with EI value: Low (Grade I, 0–0.20), moderately low (Grade II, 0.20–0.35), medium (Grade III, 0.35–0.55), moderately high (Grade IV, 0.55–0.75) and high (Grade V, 0.75–1). This study evaluates ecological conditions over YRD every five years during 1990–2010 (1990, 1995, 2000, 2005 and 2010) with the spatial resolution of 500 m × 500 m.

The key data sources used in our study include LUCC data, vegetation index data, fundamental geographical information data, as well as data from Statistical Yearbooks. Specifically, the BAI and VCI were calculated based on LUCC data. The WDI was calculated through the proportion of total river lengths, watershed areas and water resources quantity available to the total evaluation areas. As for the PLI, it was obtained from the emission of sulphur dioxide (SO_2_), chemical oxygen demand (COD) and solid waste, with the data obtained from Statistical Yearbooks. The LUCC data with a spatial resolution of 30 m were provided by the National Geomatics Centre of China (NGCC; Globeland30–2010: http://www.globalland-cover.com). A pixel-object-knowledge based approach was implemented on images from Landsat Thematic Mapper (TM), Enhanced Thematic Mapper plus (ETM+), and the HJ-1 (multispectral images from the Chinese Environmental Disaster Alleviation Satellite), with a high accuracy of over 80% [39]. There were six major land cover types, including woodland, grassland, cultivated land, water body, build-up land and unused land. The overall identification accuracy was over 95%, among which the accuracy of cultivated land reached 99%, and reached 98% for grassland, forest and built-up land [40]. The NDVI data during 1990–2010 were biweekly dataset from the Global Inventory Modeling and Mapping Studies (GIMMS NDVI3g) from the Advanced Very High Resolution Radiometer (AVHRR) [41]. Annual NDVI values were calculated using the maximum value composite (MVC) method, which minimized the effects of the solar zenith angle, cloud contamination, atmospheric and scan angels [42]. Other materials like basic geographic information, annual emission of pollutants (i.e., SO_2_ and COD) and volume of water resources were obtained from National Geomatics Center of China (NGCC), National Environment Bulletin, Water Resources Bulletin, Statistical Yearbooks and Chinese National Meteorological Center.

### 2.3. Analysis of Natural Conditions and Anthropogenic Activities

Ecological conditions were not only caused by anthropogenic activities but also natural conditions. In this paper, two natural conditions and three indicators of human activities were selected to evaluate the factors influencing the spatial variability of ecological conditions over the YRD. Elevation (Elev) and land-sea gradient (Dis_coa) were chosen to describe the natural conditions. Digital Elevation Model (DEM) data provided by space shuttle radar topography mission (SRTM) with a spatial resolution of 90 m × 90 m, were used to reflect the topography conditions over the YRD. The accuracy for DEM data at 95% confidence was 16 m, which had a strong correlation with topography, and was larger in plateau and southeast hilly terrain areas, and smaller in plains areas [43]. The urbanization rate (Urb_rate), per capita GDP (Per_gdp) and the percentage of secondary and tertiary industry employment (Per_ind) were used to reflect human activities over the YRD from the view of population and economic growth, supplied by the Statistical Yearbooks of Shanghai, and the Jiangsu and Zhejiang provinces.

Elevation differentiation and land-sea gradients are two of the basic spatial pattern features of geographic elements [33]. Due to its large difference in altitude gradient (maximum height = 1922 m) and its non-normal distribution, this paper adopts the multi-interval strip segmentation method to evaluate the influence of elevation on DEM. According to the DEM data, elevations below 100 m were separated by height intervals of 10 m, elevations between 100 m to 1000 m were separated by height intervals of 100 m, and elevations above 1000 m were considered as a strip. Therefore, the whole terrain of the YRD was separated into 20 strips, and the average values of EI were extracted for each range of elevation. For Dis_coa in the YRD, the coastline of the YRD’s mainland was buffered equidistantly to the landward side at an interval of 10 km, buffer zones from coastline to the sea were regarded as island areas and they were not considered in this paper. Consequently, we could obtain 26 strips of buffer zones and extracted an average value of EI in different strips. When it came to examining the influence of human activities on regional Eco_C, as the relative correlation between Per_gdp and Per_ind can reach 0.82 in average (*p* < 0.01), therefore, only Per_gdp was used to characterize the economic level. The average values of 25 cities’ Urb_rate, Per_gdp and Eco_C were used to classify the development mode over YRD.

Furthermore, we used the Lindeman-Merenda-Gold (LMG) metric with the “relaimpo” package in R software version 3.5.0 to make a measurement for the relative importance of each implicit factor affecting Eco_C [44]. LMG calculates the average degree of contribution of each variable in all possible sequences to the entire R^2^, thus uniquely decomposing the explained variance when predictors were correlated. This method can distinguish the contribution of different relevant predictors through a multiple linear regression model [44,45]. In this paper, these predictors include natural conditions (i.e., Elev and Dis_coa), anthropogenic activities (i.e., Urb_rate, Per_gdp and Per_ind) and the liner model with an intercept in this paper can be written as:

(1) $$\;y_{i}\;=\;\beta_{0}+Elev\beta_{1}+Dis\_coa\beta_{2}+Urb\_rate\beta_{3}+Per\_gdp\beta_{4}+Per\_ind\beta_{5}+e_{i}.$$

the response of object *i* is modelled as a linear function of regressor values Elev, Dis_coa, Urb_rate, Per_gdp and Per_ind, with unknown coefficients $\beta_{1},\cdots ,\beta_{5},$ and $e_{i}$ representing the unexplained part. In linear regression, the coefficients $\beta_{k}$, k = 0–5, are estimated by minimizing the sum of squared unexplained parts. If we denote the estimated coefficients as $\hat{\beta_{k}}$ and the fitted response values as:

(2) $$\hat{y_{i}}\;=\;\hat{\beta_{0}}+Elev\hat{\beta_{1}}+Dis\_coa\hat{\beta_{2}}+Urb\_rate\hat{\beta_{3}}+Per\_gdp\hat{\beta_{4}}+Per\_ind\hat{\beta_{5}}.$$

The coefficient of determination $R^{2}$ can be written as:

(3) $$R^{2}\;=\;\frac{Model\;SS}{Total\;SS}\;=\;\frac{\sum_{i\;=\;1}^{n}{\left(\hat{y_{i}}-\bar{y}\right)}^{2}}{\sum_{i\;=\;1}^{n}{\left(y_{i}-\bar{y}\right)}^{2}}$$

$R^{2}$ measures the proportion of variation in y that is explained by the 5 regressors in the model.

## 3. Results and Discussion

### 3.1. Spatiotemporal Variations of Eco_C in the YRD

**Figure 2:**
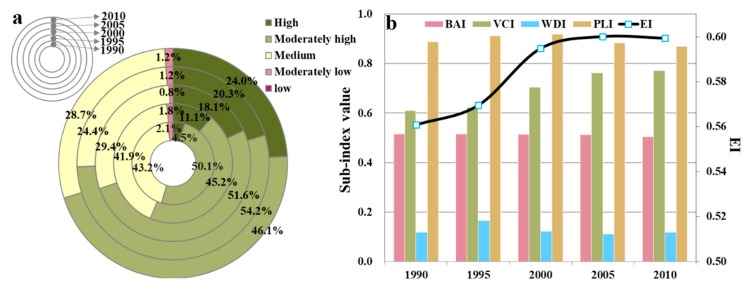
The EI area ratio of ecological conditions (**a**) and temporal variation of Eco_C and its sub-indexes (**b**) in the YRD during 1990–2010.

There existed an obvious fluctuation and an overall increasing trend for EI that ranged from 0.56 (in 1990) to 0.60 (in 2010) with an average value of 0.58 during 1990–2010 for the YRD, which was classified as “Moderately High” (Figure 2) and better than the national average. Specifically, the variation of Eco_C in YRD was the comprehensive result from the variation of each sub-index. The amount of built-up areas in the YRD, mostly urban built-up areas and rural settlements, were about 16,037 km^2^ and 28,616 km^2^ in 1990 and 2010, which increased by 78.4% and 90% respectively. This was a result of the arable land conversion [46]. During the study period, WDI and PLI values displayed an inverted “U” type trend. As the average WDI was generally low (<0.17), its impact on the EI value was relatively limited. Meanwhile, it was worth noting that the VCI value increased by 16%, which had the greatest contribution to the increase of EI value and had a significant positive effect on the improvement of Eco_C in the YRD. 

**Figure 3:**
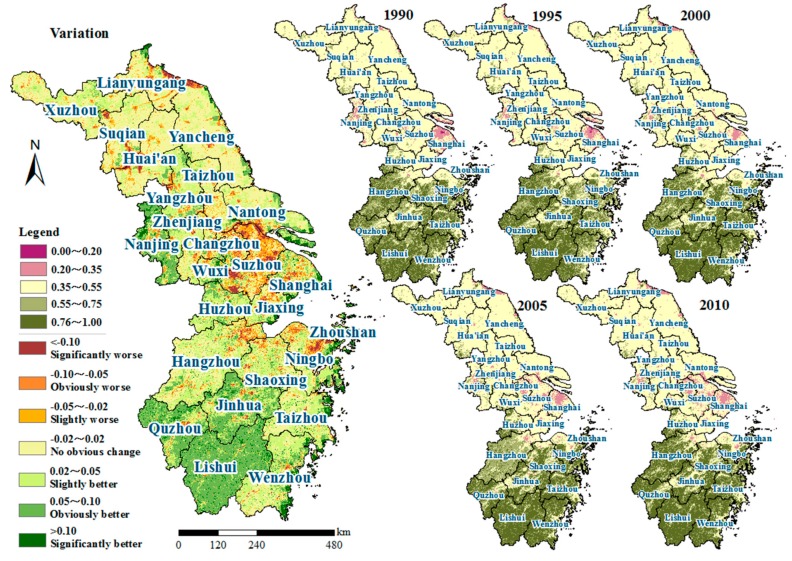
Spatial distribution of Eco_C and its variations in the YRD during 1990–2010.

There was a significant spatial distinction in Eco_C between the northern and southern areas of the YRD (Figure 3). Generally, it was divided into two parts by the demarcation belt of Shanghai-Suzhou-Wuxi-Changzhou-Nanjing whose ecological conditions were the worst, with the average EI values ranging from 0.40–0.53. The average EI values in the northern YRD were 0.53–0.57, which was lower than that in the southern areas (0.54–0.76). The overall ecological conditions in the YRD was dominated by “Medium” level, which accounted for 52.0% of the whole area, mainly distributed in Jiangsu province. The superior ecological conditions in Zhejiang province heavily relied on its high forest coverage rate (60.5%). In terms of administrative units, the highest EI value appeared in Lishui (0.76 on average) in the southern Zhejiang province, followed by Quzhou (0.72), Wenzhou (0.72), Taizhou-ZJ (0.70) and Jinhua (0.69). The Eco_C for all the cities in Jiangsu province belonged to the “Medium” level, the best Eco_C appeared in Huai’an and Suqian (0.57 on average) in the northwest regions, while the worst was in Nanjing (0.47) in the southwest area. The city with the worst Eco_C in the YRD was Shanghai and the EI difference between Shanghai and Lishui was up to 0.43–0.46.

In more detail, as shown in Figure 3, the variation of the EI values in the YRD during the study period was spatially polarized, was mainly located in the South and midlands, and presented an obvious improvement of Eco_C. The regions with EI values that got worse were mainly distributed in Suzhou, Changzhou, Zhoushan and in the northern parts of Jiangsu province such as Lianyungang. Meanwhile, the regions with little change in ecological conditions accounted for about 40% of the whole region, which were mainly concentrated in Jiangsu province. The results were consistent with the findings of Yang et al. (2015) [16]. Similarly using the YRD as an example, Yang et al. (2015) found that nearly 46.2% of the YRD including the entire southern regions, showed an obvious amelioration, whereas there was a worse trend in the northern Jiangsu province. 

### 3.2. The Variations of Eco_C across Elevation Differentiation and Land-Sea Gradient

The northern part of the YRD are plains (2.5–10 m), while the rest are complex terrains composed of mountainous (10–30 m) and medium-high and low mountains (200–500 m), which are mainly distributed in west of Taihu Lake and southwest of Tianmu Mountain [47]. The straight distance between east and west in Zhejiang province is about 450 km, while for Jiangsu province it is about 500 km.

**Figure 4:**
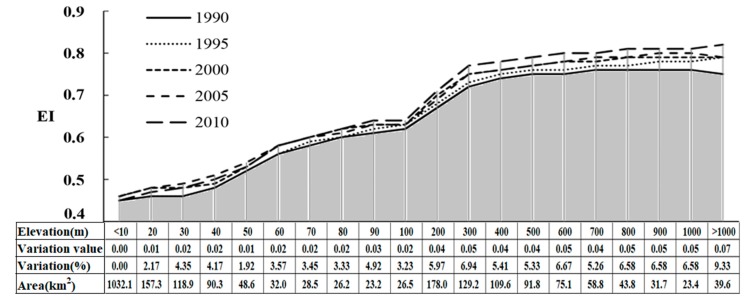
The influence of elevation differentiation on Eco_C in the YRD from 1990 to 2010.

For the YRD, EI values increased at a comprehensive level with the increase of altitude during the study period (Figure 4). It was found that the areas with elevation below 10 m had the lowest Eco_C (EI = 0.46), and the areas with elevation above 1000 m had the highest EI value (0.82). In terms of EI changes during 1990–2010, the areas with an elevation below 10 m had the minimum variation, almost with no change to the ecological conditions during the study period. Besides, the areas with an elevation above 1000 m had the maximum change, and the variation and degree of change could reach 0.07% and 9.33%. It was mainly due to the fact that elevation often affected the spatial distribution of LUCC, which in turn affected the way humans utilized land resources [48]. The low-altitude areas were usually the main regions of wide built-up land and farmland, the Eco_C was greatly affected by human activities and its values were relatively low. While high-altitude areas were dominated by woodland, grassland and little arable land, with fewer human activities on land uses, thus the Eco_C was comparatively high. In addition, the WDI value had the largest change in the areas over 1000 m, with its variation and degree of change being 0.17% and 121.43% during 1990–2010, which lead to the Eco_C in these areas having the maximum change. Moreover, the EI value increased rapidly at an altitude of 100 m to 300 m, due to the rapid increase of the BAI and VCI values, namely vegetation, woodland and grassland cover increasing significantly in this interval. 

**Figure 5:**
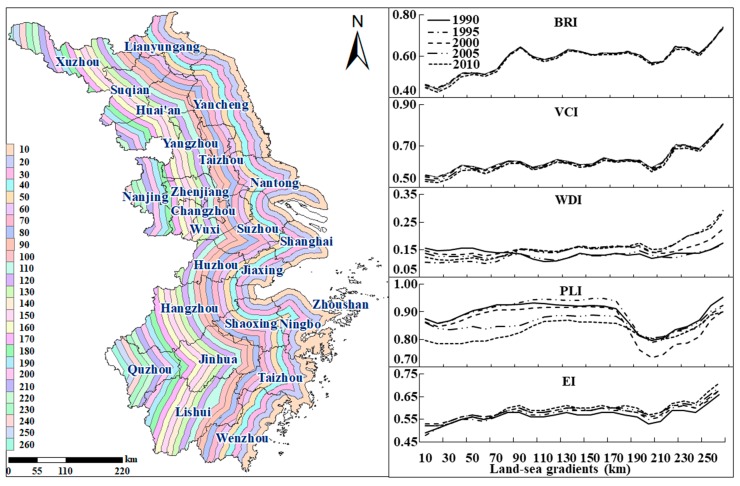
The influence of the distances to coastline on Eco_C in the YRD from 1990 to 2010.

Figure 5 illustrates the comprehensive trend of land-sea gradient from 1990 to 2010 and the accordance with the four primary indicators. It exhibited an increasing fluctuation in the comprehensive level of land-sea gradients with the distance from the coastline during the study period. The ecological conditions over 10 km region away from the coastline was the lowest (EI = 0.48), with high urbanization level, rapid economic development and intense human activities [49]. The Eco_C for the 260 km region away from the coastline was the highest (EI = 0.71). This buffer zone was mainly located in Quzhou where it had the best ecological conditions, with its BAI, VCI, WDI and PLI all at the highest levels. In addition, the Eco_C reached a more pronounced high point at 90 km (EI = 0.61), with this strip mainly possessing some water areas such as Dianshan Lake and regions with high biological abundance such as Lishui, Hangzhou, Jinhua, and Wenzhou. However, at a distance of 200 km to 210 km, there was a concave low point (EI = 0.53), as this strip passes through cities such as Nanjing, Xuzhou, Huai’an and Suqian. The relatively low Eco_C were mainly restricted by the regional environmental pollutant loads and land use types [16]. 

### 3.3. Relationship between Eco_C and Human Activities

The YRD region has experienced rapid urbanization and economic development, and now it is one of the most dynamic and promising regions in China [50]. In detail, the YRD’s urbanization level increased from 20.55% in 1980 to 74.5% in 2017, accelerating by 1.46%, which was 1.38 times the national average of 1.06% [36]. The per capita GDP of the YRD was CNY 98,912 (USD 15,479) in 2016 [35], almost double the national average (USD 8450) (OECD, 2017: http://www.oecd.org/). In addition, the tertiary (business and finance) and secondary (manufacturing) economic activities in the YRD accounted for a large proportion of this growth and for 54% and 42% of the region’s total GDP in 2016, respectively [35]. High-tech industries (medicine and electronic information) and traditional industries (computer, telecommunication equipment manufacturing and cottonocracy) were greatly developed and capitalized in the YRD [51].

Based on the average values of Urb_rate, Per_gdp and EI in 25 cities, the city development model in the YRD could be classified into seven types (Table 2). Hangzhou, with Urb_rate, Per_gdp, and EI all above the average value in the YRD, could be classified as the advanced coordinated development type. For this type, the urban expansion, economy and ecology showed a positive relationship. There were six cities that were classified as excessive development type—Shanghai, Suzhou, Nanjing, Wuxi, Changzhou, and Zhenjiang, which demonstrated a relatively high level of urbanization and economy but with compromised ecological conditions to some extent. Some cities, such as Taizhou-JS, Yancheng, Xuzhou, and Lianyungang, were at an early stage of urbanization and economic development, with the ecological conditions seriously compromised. As the urbanization level, economic development, and ecological conditions in these cities were lower than that of the regional averages, they were classified as being the primary stage of development type. The interactions among these three indicators did not have evident changes from 1990 to 2010 in nearly twenty cities. However, some other cities had evident changes from 1990 to 2010, such as Zhoushan, Suqian, Nantong, Yangzhou and Huai’an. The urbanization in Zhoushan, Nantong, Yangzhou and Suqian had experienced changes from the slow development stage to the accelerating development stage, while these urbanization processes were not coordinated with the regional GDP and ecological conditions. The development of ecological conditions lagged behind urbanization. Huai’an, marginalized by the development of the Nanjing metropolitan area, was characterized by the closed development orientation and extensive development pattern of land investment for economic scale, leading to the less significant impact from the low level of economic model on Eco_C, making its EI value fluctuate between 0.57–0.59 during 1990–2010 [52].

The results related to the urban development mode in the YRD could provide vital references for long-term policy recommendations for urbanization, environment protection and ecological conservation [53]. For those developing cities, the industrial structure should be optimized, resources should be utilized more efficiently under the base of technological progress and aimed to transform the change of the city’s economic growth model at a faster pace [2]. In addition, it could erect a local administrative management system by making full use of adjacent areas, basic legislation of the protocol and environmental standards. In this system, it is possible to use a unified early warning system, share environmental information, and adopt unitary actions and polices in order to prevent and control regional pollution [2,53]. It’s essential to harmonize urban development with the protection of ecological conditions in the YRD. 

### 3.4. Relevant Factors Affecting Eco_C Concentrations

**Figure 6:**
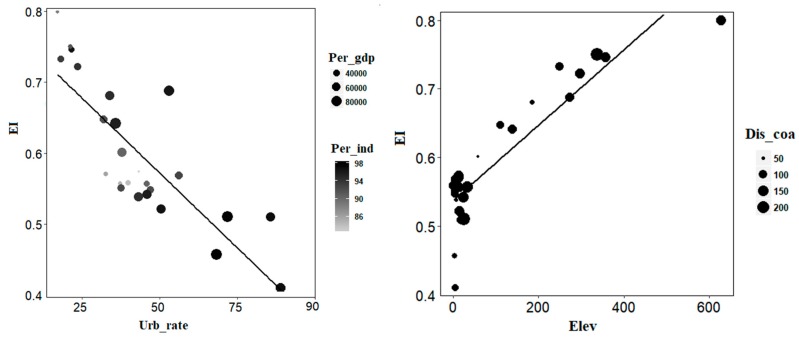
Relationship of EI with natural conditions and human activities in 2010. Eco_C: Ecological conditions; Urb_rate: Urbanization rate (%); Per_gdp: Per capita GDP (CNY); Per_ind: Percentage of secondary and tertiary industry employment (%); Elev: Elevation differentiation (m); Dis_coa: Land sea gradient (km).

For the natural conditions, there was an obviously positive relationship between Elev and EI (average *r* = 0.86) (Table 3 and Figure 6). This result was similar with that showed in Nguyen et al. (2016) [32], which concluded that the variation in altitude had the largest impact on the eco-environmental vulnerability (61.1% in total). Moreover, elevation differentiation had a significant effect on transportation, evapotranspiration and other processes that may affect Eco_C [54]. However, the relationship between land-sea gradient and EI was not apparent (*p* > 0.05), which indicated that the influence of the distance far away from the coastline on EI was very limited in the YRD. Moreover, due to all the cities in the YRD being very close to the coastline, the Dis_coa had the smallest impact. The spatial variability of Eco_C in the YRD depended strongly on Urb_rate with an average negative correlation of 0.76 (*p* < 0.01). Meanwhile, there was also a negative relationship between EI and Per_gdp (average *r* = −0.46), although the correlation was not significant enough (*p* > 0.05). The results indicated that the urban population growth and increased income had important influences on the spatial pattern of Eco_C. A similar study was also undertaken by He et al. (2017) [2], which confirmed the importance of the interaction between Eco_C and its influencing factors such as income and urbanization rate (population growth) in regional sustainable development. However, the relationship between Per_ind and EI was insignificant (*p* > 0.05), which indicated that the impact of Per_ind on EI variability was very limited. Our findings support the view that policy makers should pay more attention to the impact of income and population in framing urbanization development policies.

On the whole, the spatial Eco_C in the YRD was due to the comprehensive effects of natural conditions and human activities. Using the LMG method, the five influencing factors described in this study accounted for an average of 92.9% of the EI variability. Elev was the dominant factor contributing about 45.6%, while Urb_rate, Per_gdp, Per_ind and Dis_coa contributed about 32.5%, 9.3%, 3.5% and 2.5%, respectively (Table 3). The remaining 7.1% EI variability may be due to other natural conditions and human factors not included in this paper, such as rainfall, sunshine, land use intensities etc [17,33]. Regional ecological environment is closely related to the geographical environment, with topography playing a critical role in geomorphological, biological, and hydrological process. Altitude can affect the distribution of water and heat, and human activities can be further influenced by hydrothermal conditions [55]. In the region where plains topography and photothermal conditions are good, population distribution is concentrated, accompanying large human activities under the rapid development of economy and urbanization. Great changes have taken place in ecological conditions in these areas. On the contrary, the higher the altitude, the fewer human activities and better ecological conditions. For example, the superior ecological conditions in Zhejiang province relied heavily on its hilly terrain and high forest coverage rate (60.5%), while the worst ecological conditions were found in Shanghai where the terrain is comprised of plains and the urbanization is highly developed. With the advance of time, the impact of human activities on Eco_C was gradually strengthened, especially the dominant factor—Urb_rate, while the impacts of Per_gdp and Per_ind were weakened. This discovery was partly consistent with the findings of Sun et al. (2017) [56]. Sun et al. (2017) found that Urb_rate (population) was the dominant indicator, while Per_gdp and Per_ind were the basis and direct driving forces, respectively. However, the influence of Urb_rate on regional Eco_C was declining, while Per_gdp and Per_ind had basically maintained their effects. 

## 4. Conclusions

In this study, we investigated spatiotemporal characteristics and its determinants on ecological conditions during 1990–2010 in the YRD, China. Elevation differentiation and land-sea gradients were selected as the indicators of natural conditions that influenced the regional ecological conditions. Human activity effects were described with urbanization rate, per capita GDP and the percentage of secondary and tertiary industry employment. The results showed that there existed an obvious fluctuation and an overall increasing trend for EI that ranged from 0.56 (in 1990) to 0.60 (in 2010) with an average value of 0.58 during 1990–2010 for the YRD, which was classified into “Moderately High” (Figure 2) and better than the national average. With Shanghai-Suzhou-Wuxi-Changzhou-Nanjing as the demarcation belt, the ecological conditions in the south (mainly in Zhejiang province) were generally better than those in the north (mainly in Jiangsu province). The areas with an elevation below 10 m were relatively poor in Eco_C, while the regions above 1000 m showed the best Eco_C and had the most obvious change (9.33%) during the study period. The Eco_C over 10 km away from the coastline was the lowest and showed an increasing trend with the distance from the coastline, while a more pronounced high point appeared at 90 km (EI = 0.61) and a concave low point (EI = 0.53) appeared at a distance of 200–210 km to the coastline, which were inseparable from human activities. Besides this, the elevation was a key factor that effected the spatial patterns of ecological conditions (*r* = 0.86). Ecological conditions increased with the altitude and the distance from the coastline during 1990–2010. In addition, for the urban development mode in YRD, all 25 cities could be separated into seven types. The interactions among human activities and ecological conditions did not have evident changes in nearly twenty cities, while an obvious change appeared in Zhoushan, Suqian, Nantong, Yangzhou and Huai’an. Further analysis indicated that the five selected factors, including two indicators of natural conditions and three indicators of human activities, could explain 92.19% in average of the ecological condition variability. Elevation differentiation had a remarkably positive correlation with EI, whereas the urbanization rate had a negative impact (average *r* = −0.76) on regional ecological conditions, followed by per capita GDP (average *r* = −0.46).

This paper described the ecological conditions in the YRD with a synthetic index. However, the explaining factors illustrated in this study could not express the variations in ecological conditions completely. Given the complexity of natural environment, comprehensive analysis including more anthropogenic and natural factors such as rainfall, sunshine and human activities on land use intensities are needed in future research work.

## Figures and Tables

**Table 1:** Calculation methods of EI and its sub-indexes.

Index	Formula *	Description
BAI	BAI = A1 × (0.35 × A_woo_ + 0.21 × A_gra_ + 0.28 × A_wat_ + 0.11 × A_cul_ + 0.04 × A_bui_ + 0.01 × A_un_)/A_YRD_	*A_woo_*, *A_gra_*, *A_cul_*, *A_wat_*, *A_bui_*, and *A_un_* are the areas of woodland, grassland, cultivated land, water body, build-up land, and unused land, respectively. *A_YRD_* is the area of the YRD. *L_riv_*, *A_lak_* and *V_wat_* are the river lengths, lake areas and the volumes of water resources in the YRD, respectively. *A_SO2_*, *A_COD_* and *A_was_* denote annual emissions of SO_2_, COD and solid waste in each city, and *P* is annual average precipitation.
VCI	VCI = A2 × NDVI_max_	
WDI	WDI = 1/3 × A3 × L_riv_/A_YRD_ + 1/3 × A4 × A_lak_/A_YRD_ + 1/3 × A5 × V_wat_/A_YRD_	
PLI	PLI = 0.4 × (100-A6 × A_SO2_/A_YRD_) + 0.4 × (100-A7 × A_COD_/P) + 0.2 × (100-A8 × A_was_/A_YRD_)	
**EI**	**EI = 0.25 × BAI + 0.3 × VCI + 0.2 × WDI + 0.25 × PLI**	

* A1–A9 stand for the corresponding normalized coefficient of each factor. Where *A*, *A_max_* and *A_min_* separately denote the actual value, maximum value and minimum value.

**Table 2:** Urban development mode in the YRD during 1990–2010.

Types	Urbanization Level	Per Capita GDP	EI	Cities	Descriptions
**Advanced coordination type**	+	+	+	Hangzhou	Healthy city development process, urbanization has a positive relationship with economic development and ecological conditions.
**Excessive development type**	+	+	−	Shanghai, Suzhou, Nanjing, Wuxi, Changzhou, Zhenjiang, Zhoushan (1990)	High urbanization level and economic development, but with compromised ecological conditions.
**Fast expanding development type**	+	−	−	Zhoushan (1995), Suqian (2005/2010), Nantong (1995/2000/2010), Yangzhou (2005/2010)	Economic development could not keep up with the rate of urban expansion, and the expansion also brought negative impacts on ecological conditions.
**Steady development type**	−	+	+	Ningbo, Shaoxing, Zhoushan (2010)	Fast economic development with good ecological conditions, but low urbanization level
**Lagging development type**	−	+	−	Jiaxing, Zhoushan (2005)	Urban expansion could not keep up with economic development, and ecological conditions have been compromised.
**Primary stage development type**	−	−	+	Wenzhou, Jinhua, Quzhou, Lishui, Huzhou, Taizhou, Huai’an (1990/2000), Suqian (1990/1995),	Low urbanization and economic development level, and uncompromised ecological conditions.
**Inferior development type**	−	−	−	Taizhou (JS), Yancheng, Xuzhou, Lianyungang, Zhoushan (2000), Huai’an (1995/2005/2010), Suqian (2000), Nantong (1990/2005), Yangzhou (1990/1995/2000)	Experiencing acceleration of urbanization and economic development, as well as the deterioration of ecological conditions.

Note: “+” means above or close to YRD average level, “−” was lower than the average; in the “Representative cities”, the city without “()” means exists in 1990–2010, the year in brackets mean the type of that year.

**Table 3:** The correlation coefficients (r) among EI and its influencing factors during 1990–2010. The proportion of the EI variance explained by the influencing factors with LMG metric was also illustrated in brackets.

	Urb_rate	Per_gdp	Per_ind	Elev	Dis_coa	Total_Con
Eco_C_1990	−0.74 ** (28.60%)	−0.64 ** (18.50%)	−0.33 (8.30%)	0.80 ** (42.40%)	0.19 (2.20%)	92.11%
Eco_C_1995	−0.70 ** (35.10%)	−0.39 (7.40%)	−0.21 (3.20%)	0.85 ** (51.20%)	0.21 (3.30%)	93.73%
Eco_C_2000	−0.76 ** (35.30%)	−0.40 (7.50%)	−0.17 (2.30%)	0.88 ** (52.90%)	0.18 (1.90%)	94.35%
Eco_C_2005	−0.80 ** (36.00%)	−0.50 * (10.40%)	−0.19 (2.60%)	0.85 ** (47.40%)	0.28 (3.60%)	90.99%
Eco_C_2010	−0.82 ** (39.90%)	−0.39 (6.40%)	−0.12 (2.20%)	0.89 ** (48.80%)	0.24 (2.70%)	93.36%
Average	−0.76 ** (32.51%)	−0.46 * (9.30%)	−0.20(3.45%)	0.86 ** (45.13%)	0.22 (2.54%)	92.91%

** *p* < 0.01 * *p* < 0.05. Eco_C: Ecological conditions; Urb_rate: Urbanization rate (%); Per_gdp: Per capita GDP (CNY); Per_ind: Percentage of secondary and tertiary industry employment (%); Elev: Elevation differentiation (m); Dis_coa: Land sea gradient (km); Total_Con: Total contribution.
